# Successful mesenchymal stem cell treatment of leg ulcers complicated by Behcet disease: A case report and literature review: Erratum

**DOI:** 10.1097/MD.0000000000010670

**Published:** 2018-04-27

In the article, “Successful mesenchymal stem cell treatment of leg ulcers complicated by Behcet disease: A case report and literature review”,^[1]^ which appeared in Volume 97, Issue 16 of *Medicine*, the legend for Figure 1 should be:

Figure 1. Leg Ulcer biopsy. Small vessel leukocytoclastic vasculitis (H&E, 200x).
